# An Ultrasensitive Electrochemical Sensor Using Banana Peel Activated Carbon/NiFe_2_O_4_/MnCoFe-LDH Nanocomposites for Anticancer Drug Determination

**DOI:** 10.1021/acsomega.4c02460

**Published:** 2024-06-12

**Authors:** Nevin Erk, Wiem Bouali, Asena Ayse Genc, Qamar Salamat, Mustafa Soylak

**Affiliations:** † Faculty of Pharmacy, Department of Analytical Chemistry, Ankara University, Ankara 06560, Turkey; ‡ The graduate school of the health sciences, Ankara University, Ankara 06110, Turkey; § Faculty of Sciences, Department of Chemistry, Erciyes University, Kayseri 38039, Turkey; ∥ Technology Research & Application Center (TAUM), Erciyes University, Kayseri 38039, Turkey; ⊥ Turkish Academy of Sciences (TUBA), Cankaya, Ankara 06670, Turkey

## Abstract

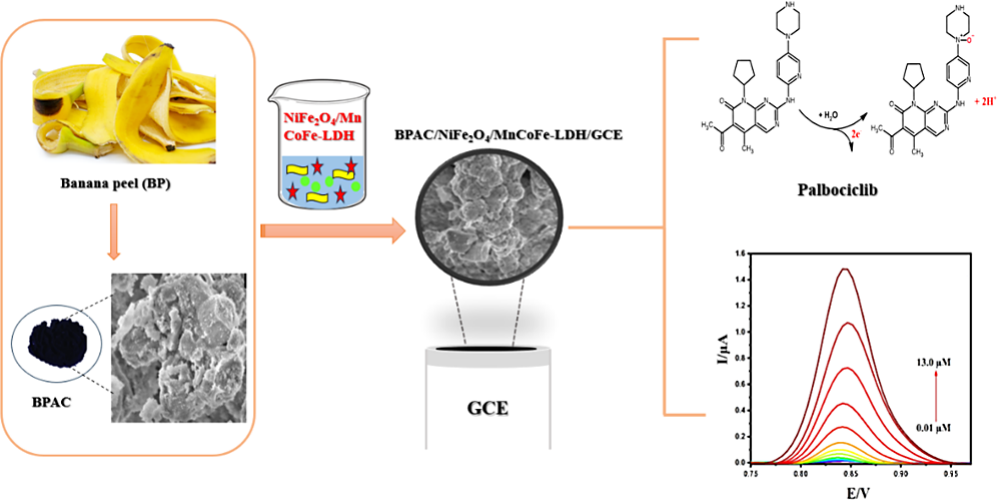

In the current study, we report the synthesis of a novel composite material composed of banana peel activated carbon (BPAC), nickel iron oxide (NiFe_2_O_4_), and manganese cobalt iron layered double hydroxide (MnCoFe-LDH) to create a high-performance electrochemical sensor to detect Palbociclib (PLB). The composite was successfully immobilized on a glassy carbon electrode (GCE) surface to create a modified electrode. The performance of the electrode was thoroughly evaluated, considering parameters such as electroactive surface areas (ESA), electron transfer rate constant (k^0^), and exchange current density (j_0_). The developed BPAC/NiFe_2_O_4_/MnCoFe-LDH/GCE exhibited a wide linear dynamic range of 0.01–13.0 μM for PLB concentration, accompanied by a detection limit at a low level (3.5 nM). Furthermore, it can be applied to the determination of PLB in human urine and pharmaceutical samples with excellent recoveries (98.5–102.9%) and RSD values lower than 3%, establishing its potential for precise PLB determination in pharmaceutical and biological samples. This research contributes to the advancement of electrochemical sensor technology for the detection of important anticancer drugs in real-world applications.

## 1 Introduction

Palbociclib, a groundbreaking cyclin-dependent kinase 4/6 (CDK4/6) inhibitor, received approval from the U.S. Food and Drug Administration (FDA) in 2015, signifying a pivotal advancement in the therapeutic landscape for metastatic breast cancer distinguished by hormone receptor-positive (HR+) and human epidermal growth factor receptor 2-negative (HER2-) status.^1^ Particularly distinguished as a first-line treatment when administered alongside aromatase inhibitors (AIs) or fulvestrant, Palbociclib has reshaped treatment strategies for advanced breast cancer.^2^ The recommended starting dosage of PLB, at 125 mg once daily following a’3 weeks on and 1 week off’ schedule, underscores its precision in aligning treatment regimens with patient needs.^3^ Accurate quantification of PLB is instrumental in elucidating its pharmacokinetic properties, optimizing therapeutic regimens, and ensuring patient safety.^4^ Furthermore, developing sensitive and selective analytical methods for PLB detection is pivotal for pharmacological studies, allowing for a comprehensive understanding of its behavior in biological matrices. Various techniques have been reported for the determination of PLB, as a single component or in combinations with other drugs, including liquid chromatography–tandem mass spectrometry (LC–MS/MS),^5^ high-performance liquid chromatography (HPLC),^6^ spectrofluorimetric,^7^ UV spectrophotometric,^8^ and electrochemical^9^ methods.

Electrochemical sensors, particularly those employing modified GCEs, have emerged as powerful tools for the determination of anticancer drugs, offering a combination of high sensitivity, selectivity, real-time analysis, simplicity, and portability.^10^ GCEs modified with one or more agents, have garnered considerable attention owing to their ability to form well-defined surfaces, exhibit low background currents, operate across a wide range of potentials, and maintain chemical inertnes.^11^

The utilization of agricultural waste-derived banana peel activated carbon (BPAC) has gained significant traction in scientific research, representing a sustainable and easily accessible biomass resource.^12^ Banana peels, constituting a substantial 40% of the total weight of bananas and regarded as one of the largest agricultural wastes, have emerged as valuable precursors owing to their abundance, low cost, and ease of harvest.^13^ To date, diverse investigations have delved into the exploration of banana trees, showcasing their versatile applications. These include studies on porous carbon materials derived from banana peels for battery applications,^14^ the adsorption potential of banana peels for gases,^15^ and the use of activated banana peels in electrochemical sensors.^16^ The success of a modified electrode incorporating sulfur-doped banana peel-derived activated carbon in supercapacitor applications underscores the versatility of this eco-friendly composite.^17^

Magnetic nanoparticles (NPs), commonly formulated as MFe_2_O_4_ (where M = Fe, Ni, Co, Cu, Mn, etc.), stand out as prominent materials in medicine, biochemistry, biotechnology, and heavy metal removal.^18^ Within the realm of these magnetic nanoparticles, NiFe_2_O_4_ nanoparticles have emerged as a focal point in sensor technology due to their notable biocompatibility, exceptional chemical stability, elevated mechanical hardness, electromagnetic prowess, facile preparation, and pronounced adsorption capabilities.^19^ Additionally, NiFe_2_O_4_ NPs exhibit a substantial surface area and minimal mass transfer resistance.^20^

Recent advancements in nanotechnology have ushered in a new era of material synthesis, exemplified by the production of layered double hydroxides (LDHs) imbued with distinctive properties.^21^ Their emergence as a promising category of nonprecious bifunctional electrocatalysts in alkaline electrolyte solutions stems from their unique 2D structure, offering substantial surface areas, tunable compositions, and abundant availability. The strategic introduction of various transition metals with mixed valence into LDH formulations has shown tremendous potential in elevating the catalytic activities of LDHs.^22^ Recent investigations have specifically highlighted the notable promise of CoFe-based LDHs, elucidating a robust synergistic effect between Co and Fe ions.^23^ Additionally, studies incorporating cobalt ferrite doping with metal ions, such as Mn^2+^, have garnered significant attention, particularly in applications of sensor technology and biomedical applications.^24^

Therefore, the incorporation of NiFe_2_O_4_ onto the MnCoFe-LDH surface, coupled with banana peel activated carbon (BPAC) to create a composite, is anticipated to yield an excellent specific surface area, heightened redox activity, and elevated conductivity.

Based on the thoughts above, in the present work, a pioneering and effective electrochemical sensor has been developed for the sensitive detection of the crucial anticancer agent Palbociclib (PLB) using a low-cost, and environment-friendly composite (BPAC/NiFe_2_O_4_/MnCoFe-LDH). The electrochemical sensing platform (BPAC/NiFe_2_O_4_/MnCoFe-LDH-modified GCE) exhibited remarkable electrocatalytic prowess, sensitivity, and selectivity toward PLB.

## 2 Experimental Section

### 2.1 Chemicals and Apparatus

The Electronic Supporting Information contains information on chemicals and apparatus.

### 2.2 Synthesis of the BPAC/NiFe_2_O_4_/MnCoFe-LDH Nanocomposite

Synthesis of banana peel activated carbon and NiFe_2_O_4_ nanoparticles was carried out as described in the Supporting Information. The synthesis procedure of BPAC/NiFe_2_O_4_/MnCoFe-LDH nanocomposite is outlined as follows:^25^ First, 0.5 g of synthesized NiFe_2_O_4_ was dispersed in 15 mL of distilled water. After that, 10 mmol Mn (NO_3_)_2_, 4H_2_O, 20 mmol Fe (NO_3_)_3_·9H_2_O, and 2.5 mmol Co (NO_3_)_2_·6H_2_O were added to the previous mixture at room temperature, the components were combined and stirred for 20 min to achieve a homogeneous solution. Subsequently, 40 mmol of NaF was added to the resulting solution (referred to as Mixture A). Concurrently, 80 mmol of urea was dissolved in 20 mL of deionized water and stirred for 10 min to form another solution (referred to as Mixture B). Afterward, Mixture B was added into Mixture A drop by drop, with continuous stirring maintained for an additional 20 min. Subsequently, 0.5 g of prepared BPAC was added to the previous solution, and the mixture was stirred for an additional 10 min. The resulting mixture was transferred into a 60 mL Teflon container, which was then positioned in an autoclave. The autoclave was subsequently placed in an oven and heated at 120 °C for a duration of 10 h. Following the completion of the heating process, the autoclave was allowed to cool to room temperature. The resulting product was subsequently washed sequentially with deionized water and ethanol to remove impurities. Finally, the washed product was dried in an oven at 70 °C for a period of 6 h to obtain the final product.

### 2.3 Construction of Modified Electrodes and Electrochemical Assessments

To prepare the GCE, the bare electrode underwent polishing with alumina slurries and was then sonicated in a mixture of equal volumes of deionized water and ethanol for 5 min.

The composite was dispersed in deionized water and subjected to ultrasonication for 1 h. Subsequently, the resulting BPAC/NiFe_2_O_4_/MnCoFe-LDH nanocomposite suspension was utilized for the surface modification of GCE. A 7.0 μL amount of the BPAC/NiFe_2_O_4_/MnCoFe-LDH (1.5 M) suspension was cast onto the GCE surface and subjected to drying for 20 min utilizing an infrared heat lamp.^26^

All electrochemical investigations were conducted utilizing BPAC/NiFe_2_O_4_/MnCoFe-LDH/GCE as the working electrode, platinum rod as the counter electrode, and Ag/AgCl saturated solution serving as the reference electrode. The EIS and CV procedures were executed in a solution of [Fe(CN)_6_]^3–/4–^ (5.0 mM) and KCl (0.1 M). CV measurements were examined within a potential range from −0.5 to 1.0 V at a scan rate of 50 mV/s, and EIS studies were conducted across a frequency range of 10 kHz to 0.1 Hz at a potential of 0.1 V. In addition, DPV with a modulation amplitude and time of 0.05 V and 0.01 s, and a step potential of 0.005 V, was employed for the determination of PLB in B-R buffer at pH 2.0.^27^

### 2.4 Processing of Actual Samples

The novel sensor in this study was used for the precise determination of PLB across human urine and pharmaceutical tablets. The real samples were prepared according to our previous procedure.^28^

## 3 Results and Discussion

### 3.1 Characterizations of BPAC/NiFe_2_O_4_/MnCoFe-LDH

**Figure 1:**
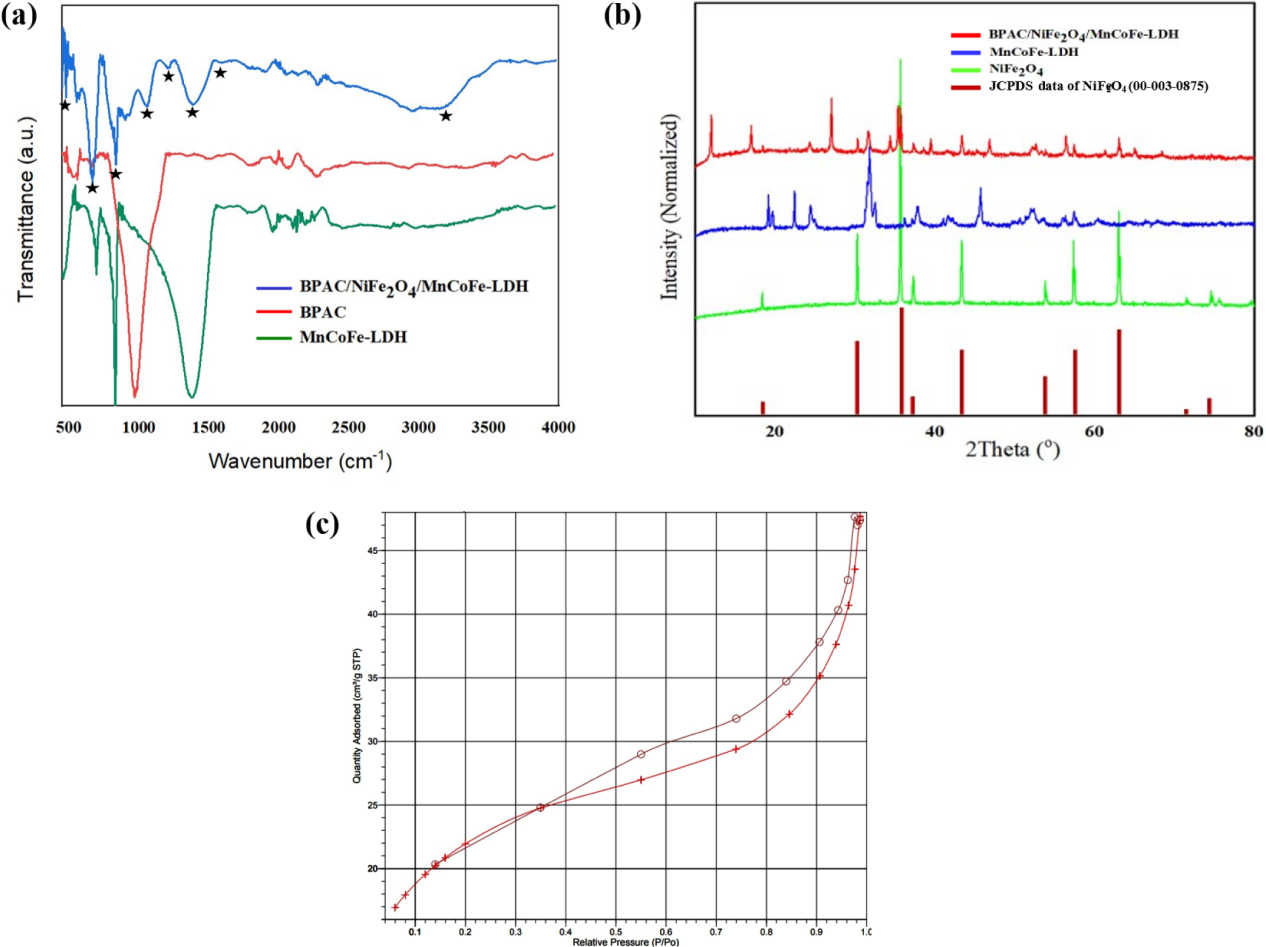
(a) FT-IR spectra of the MnCoFe-LDH, BPAC, and BPAC/NiFe_2_O_4_/MnCoFe-LDH nanocomposite. (b) XRD patterns of NiFe_2_O_4_, MnCoFe-LDH, BPAC/NiFe_2_O_4_/MnCoFe-LDH nanocomposite, and JCPDS data of NiFe_2_O_4_. (c) N_2_ adsorption/desorption study of the BPAC/NiFe_2_O_4_/MnCoFe-LDH nanocomposite.

The binding sites, functional groups, degree of crystallinity, morphology, surface area, pore diameter of the materials, the elemental composition, and the chemical analysis of the nanocomposite and its components were evaluated by applying the FT-IR, XRD, SEM, SEM-EDX, and BET instrumental analysis. In the FT-IR spectrum (Figure 1a), the broad absorption peak observed at 3175 cm^–1^ unequivocally corresponds to the stretching vibrational mode of −OH groups. The absorption band at 1617 cm^–1^ is attributed to the presence of the NO_3_^–^ anion situated between the layers of MnCoFe-LDH, and the wide absorption band detected at 1401 cm^–1^ is ascribed to the bending vibration of H_2_O molecule and −OH groups situated interlayer between hydrotalcite layers, as illustrated in the accompanying picture.^29^ The −OH groups serve to counterbalance the positively charged ions (Mn^2+^, Co^2+^, and Fe^3+^) within the structural framework.^30^ Additionally, the peak observed at 1233 cm^–1^ corresponds to the C–N stretching bond. The N–O group exhibits an absorption peak at 1067 cm^–1^. Moreover, the absorption signals observed below 1000 cm^–1^ (851, 680, and 466 cm^–1^) are indicative of the vibration and stretching modes associated with the metal and oxygen lattice within the hydrotalcite-like lattice (M–O, M–O–M, and O–M–O bond), thus confirming the successful synthesis of MnCoFe-LDH.^31^ Furthermore, two absorption bands at around 570 and 588 cm^–1^, correspond to the octahedral and tetrahedral sites of positive ions of NiFe_2_O_4_, respectively.^32^

The X-ray diffraction (XRD) analysis (Figure 1b) was conducted to verify the phase purity and crystallinity of the synthesized materials, including NiFe_2_O_4_, MnCoFe-LDH, the BPAC/NiFe_2_O_4_/MnCoFe-LDH nanocomposite, and JCPDS data for NiFe_2_O_4_. The distinctive peaks observed at 24.4°, 37.3°, and 46.8° can be attributed to CoFe-LDH (JCPDS PDF#50–0235), while the diffraction peak at 2θ of 52.6°, aligns with literature data for Mn-LDH (JCPDS No. 10-144).^33^ Regarding the presence of NiFe_2_O_4_ in the nanocomposite, the diffractogram indicates different reflection planes indexed as 30.27°, 35.74°, 37.28°, 43.47°, 47,30°, 53.88°, 57.55°, 62.72°, 71.40°, and 74.67° which indicates the spinel cubic structure of NiFe_2_O_4_^34^ (JCPDS No. 00-003-0875). The most intense XRD peak at 2θ = 35.73 corresponds to the plane of NiFe_2_O_4_ (inverse spinel structure).^35^

Nitrogen adsorption analyses, employing the Brunauer–Emmet–Teller (BET) and Barret–Joyner–Halenda (BJH) methods, were conducted using a Micromeritics apparatus to ascertain the surface area and pore diameter of the materials (Figure 1c). This instrumentation features dual independent vacuum systems: one designated for sample preparation and another for analysis, enabling concurrent treatment and analysis of distinct samples. The BET analysis proceeded in two phases: initial sample treatment (degassing) followed by sample analysis. Degassing, the preliminary step, involves purging the sample to eliminate contaminants, followed by heating and vacuum exposure. This preparatory phase is pivotal, as solid materials tend to absorb moisture and impurities from the ambient atmosphere, potentially compromising data reliability and equipment integrity. Subsequently, the treated samples underwent analysis to determine crucial parameters such as surface area, pore diameter, and volume.

The prepared BPAC/NiFe_2_O_4_/MnCoFe-LDH nanocomposite exhibits a type IV isotherm with H_3_ hysteretic loop, indicative of mesoporous materials composed of plate-like particle aggregates. According to the BET analysis results, the surface area of the BPAC/NiFe_2_O_4_/MnCoFe-LDH nanocomposite is estimated to be 69.645 m^2^/g. Furthermore, the pore volume and pore size are determined to be 0.073774 cm^3^/g and 42.3709 Å, respectively.

**Figure 2:**
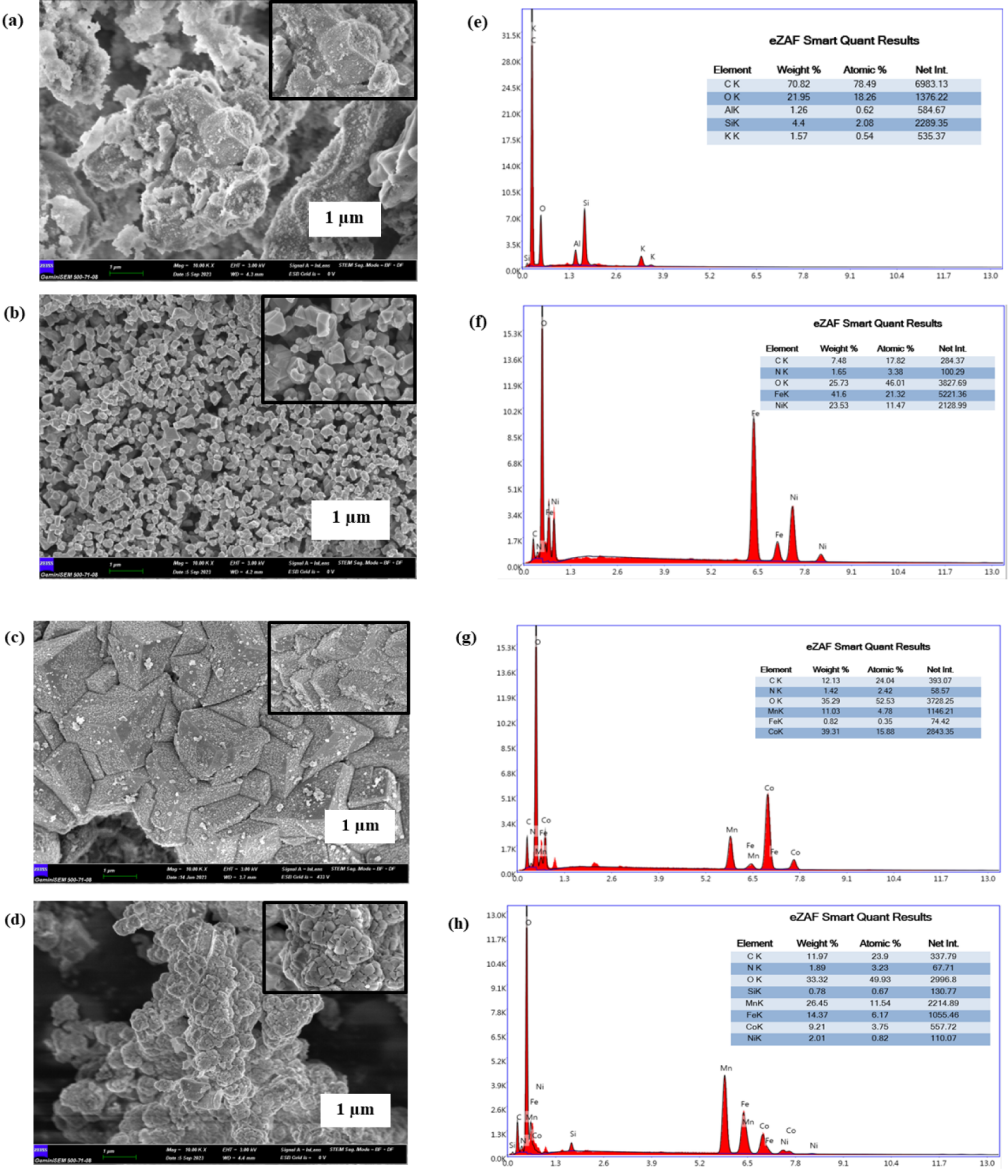
SEM images and energy-dispersive X-ray (EDX) of (a,e) BPAC, (b, f) NiFe_2_O_4_, (c, g) MnCoFe-LDH, and (d, h) BPAC/NiFe_2_O_4_/MnCoFe-LDH nanocomposite.

The size and morphology of the NiFe_2_O_4_, MnCoFe-LDH, and BPAC/NiFe_2_O_4_/MnCoFe-LDH composite were assessed using scanning electron microscopy (SEM), as illustrated in Figure 2. These images reveal particles with uniform sizes and well-defined shapes. At first, activated carbons were obtained from banana peels. The surface of the material exhibits numerous pores, while the external surface of the banana peel displays pores of varying sizes and shapes. The relatively smooth external surface of the banana nanoparticles indicates their composition as numerous small primary nanoparticles. The aggregation of these primary nanoparticles gives rise to numerous intra-aggregated pores, thereby contributing to a high microporous volume (Figure 2a). The synthesized NiFe_2_O_4_ in Figure 2b exhibits a hexagonal bipyramidal shape, with particle sizes of approximately 200 nm. Figure 2c indicates typical SEM images of MnCoFe-LDH, characterized by crystal-shaped micro flower-like structures. The SEM images revealed that the surface of the LDH exhibited a relatively rough texture, characterized by numerous minor nanofolds and shallow channels. This surface morphology contributes to the generation of a large surface area. In Figure 2d, the SEM images of the BPAC/NiFe_2_O_4_/MnCoFe-LDH nanocomposite displayed, reveal even distribution of BPAC/NiFe_2_O_4_ crystals on the surface of MnCoFe-LDH. These findings provide strong evidence for the successful synthesis of the BPAC/NiFe_2_O_4_/MnCoFe-LDH nanocomposite. SEM-EDX investigation was conducted to validate the elemental composition of the BPAC, NiFe_2_O_4_, MnCoFe-LDH, and BPAC/NiFe_2_O_4_/MnCoFe-LDH compsite.

### 3.2 Electrochemical Behavior of the Sensing Platform

**Figure 3:**
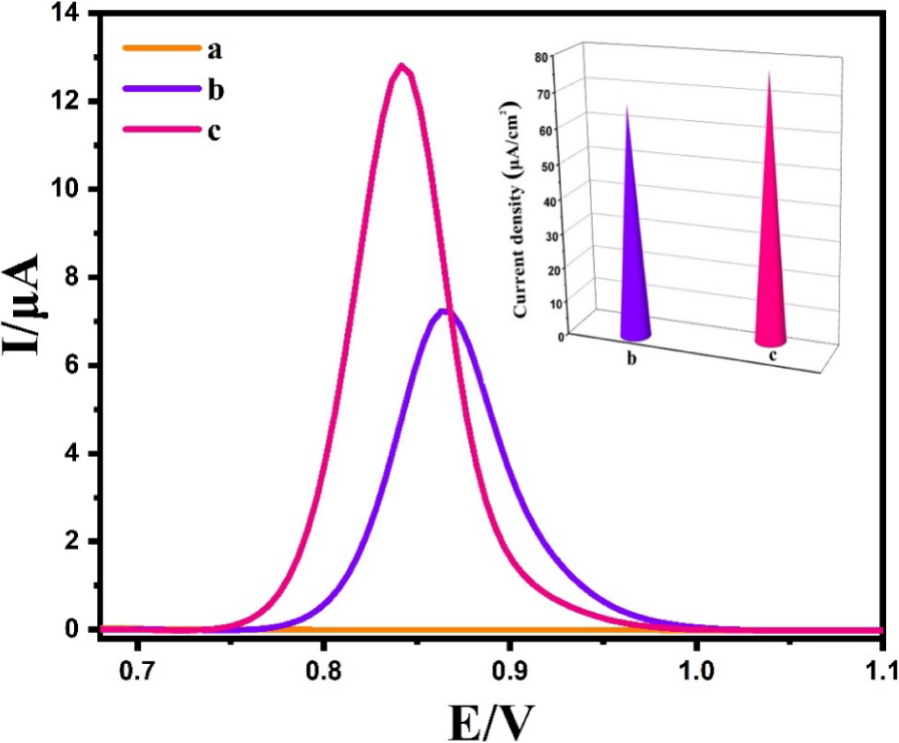
DPVs of blank (a), bare/GCE (b), and BPAC/NiFe_2_O_4_/MnCoFe-LDH/GCE (c) in 0.1 M BR solution (pH 2.0) containing 0.1 mM PLB.

To investigate the electrochemical response of PLB at both the GCE and BPAC/NiFe_2_O_4_/MnCoFe-LDH/GCE, DPV responses were recorded in a BR buffer containing 0.1 mM PLB (Figure 3). Experiments were conducted in the absence and presence of PLB in a BR electrolyte solution (0.1 M, pH 2). A baseline voltammogram of BPAC/NiFe_2_O_4_/MnCoFe-LDH/GCE indicated the absence of an oxidation peak in the absence of PLB, confirming the inert nature of the used composite. Upon the addition of 0.1 mM PLB, the bare GCE displayed an oxidation peak of 7.20 μA (0.86 V vs Ag/AgCl). In contrast, the modified electrode exhibited a well-defined oxidation peak (0.84 V vs Ag/AgCl), accompanied by a significantly higher current response of 12.8 μA. These results signify a notable improvement in the electrochemical reactivity of PLB on BPAC/NiFe_2_O_4_/MnCoFe-LDH/GCE, evidenced by the negative shift in the potential of PLB oxidation and the concurrent increase in peak current. Furthermore, employing the acquired voltammograms, the current density was calculated by dividing the obtained current by the surface area of the working electrode. Analyses demonstrated that the modified electrode displayed a higher current density of 75.3 μA cm^–2^ compared to the bare electrode, which recorded a current density of 65.4 μA cm^–2^. These results underscores the BPAC/NiFe_2_O_4_/MnCoFe-LDH’s electrocatalytic activity for the oxidation of PLB, offering valuable insights into its potential application in electrochemical sensing.

**Figure 4:**
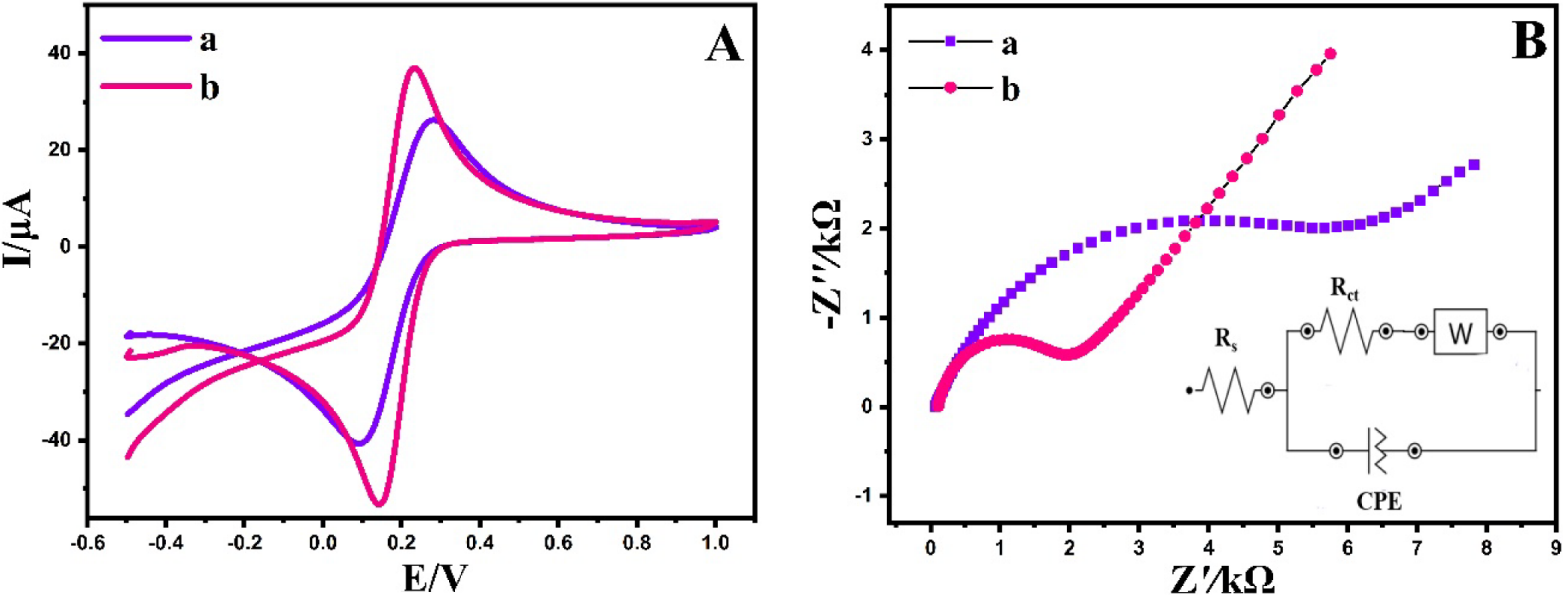
CVs (A), and EIS curves (B) of the bare electrode (a) and BPAC/NiFe_2_O_4_/MnCoFe-LDH/GCE (b) in a solution of [Fe(CN_6_)]^3–/4–^ and KCl. The inset is the equivalent circuit fitting (R_s_ electrolyte solution resistance, R_ct_ element of interfacial electron transfer resistance, CPE constant phase element, W Warburg impedance resulting from the diffusion of ions).

Moreover, the electrochemical characteristics of various electrodes were evaluated in [Fe(CN)_6_]^3–/4–^ (5.0 mM) and 0.1 M KCl by performing a cyclic voltammetry test while maintaining a constant scan rate of 50.0 mV/s (Figure 4). As depicted in Figure 4A, the unmodified electrode exhibited distinct peak currents for the [Fe(CN)_6_]^3–/4–^, characterized by a peak potential difference (Δ*E*_p_) of 180 mV (curve a). Following the modification of the electrode with the BPAC/NiFe_2_O_4_/MnCoFe-LDH composite, the cyclic voltammogram of the [Fe(CN)_6_]^3–/4–^ redox couple displayed an augmentation in both anodic and cathodic peak currents, accompanied by a reduction in Δ*E*_p_ to 80 mV (curve b). The enhanced electrochemical properties observed in BPAC/NiFe_2_O_4_/MnCoFe-LDH/GCE can be explained by the expanded electroactive surface area and heightened conductivity arising from the integration of BPAC, NiFe_2_O_4_, and MnCoFe-LDH into the composite.

The CV test of both the bare and BPAC/NiFe_2_O_4_/MnCoFe-LDH/GCE was conducted in a solution of [Fe(CN)_6_]^3–/4–^ and KCl at various scan rates (10–300 mV/s). With an increment in the scan rate, the I_pa_ linearly increased in both electrodes (Figures S1 and S2). The electroactive surface areas (ESA) of the electrodes were determined by utilizing the slope of the cathodic peak current versus the square root of the scan rate, and the Randles–Sevick equation (S1). The ESA of BPAC/NiFe_2_O_4_/MnCoFe-LDH/GCE was calculated to be 0.17 cm^2^, surpassing that of the bare electrode (0.11 cm^2^). This finding suggests that the BPAC/NiFe_2_O_4_/MnCoFe-LDH-modified GCE is poised to exhibit superior electrocatalytic performance compared to the bare GCE.

To investigate the electrochemical properties of the BPAC/NiFe_2_O_4_/MnCoFe-LDH/GCE, the EIS method was employed. EIS recognized as an efficient approach for probing electrode interface properties, relies on the value of the charge-transfer resistance (R_ct_) to govern the kinetics of electron transfer for the analyte at the interface of the electrode, thereby highlighting the bonding of each substrate to the surface of the electrode. As illustrated in Figure 4B, the bare GCE displays a significant semicircular section at high frequencies, indicative of a high charge transfer resistance, with an R_ct_ value of 5.9 kΩ (curve a). This high R_ct_ value is associated with low charge and mass transfer rates at the surface of the unmodified electrode. Upon the incorporation of the BPAC/NiFe_2_O_4_/MnCoFe-LDH composite at the GCE surface, a notably lower R_ct_ value of 2.1 kΩ was achieved (curve b). The diminished R_ct_ value for the BPAC/NiFe_2_O_4_/MnCoFe-LDH-modified electrode signifies an improved electron transfer rate compared to the bare electrode, aligning well with the cyclic voltammetry (CV) results. These results underscores the positive impact of the BPAC/NiFe_2_O_4_/MnCoFe-LDH composite on enhancing the electrochemical performance of the modified electrode.

Furthermore, the electron transfer rate constant (k^0^) and the exchange current density (j_0_) for the bare GCE and BPAC/NiFe_2_O_4_/MnCoFe-LDH/GCE were calculated from the EIS data using the eqs S2 and S3 and listed in Table 1. The determination of heterogeneous electron transfer rate constant (k^0^) values was essential to evaluate the kinetic feasibility of the redox pair. A system characterized by a low k^0^ value signifies a more prolonged time scale to reach equilibrium compared to a system with a high k^0^ value, which achieves equilibrium more rapidly. The obtained k^0^ values elucidate that the modified electrode exhibits a higher k^0^ value (15.1 × 10^–5^ cm s^–1^) compared to the bare electrode (8.2 × 10^–5^ cm s^–1^). This difference implies that the modified electrode facilitates faster electron transfer kinetics, underscoring its enhanced electrochemical performance.

**Table 1:** Electrochemical Parameters Acquired through CV and EIS Assessments Conducted on Various Working Electrodes

electrode	Δ*E*_p_(V)	ESA (cm^2^)	*k*^0^ (cm s^–1^)	j_0_(A cm^2^)
GCE	0.18	0.11	8.2 × 10^–5^	3.9 × 10^–5^
BPAC/NiFe_2_O_4_/MnCoFe-LDH/GCE	0.08	0.17	15.1 × 10^–5^	7.2 × 10^–5^

The determined exchange current density (j_0_) of 7.2 × 10^–5^ (A cm^–2^) for the composite modified electrode, BPAC/NiFe_2_O_4_/MnCoFe-LDH/GCE, surpassed that of the bare electrode, registering at 3.9 × 10^–5^ (A cm^–2^). This noteworthy enhancement can be attributed to the composite electrode’s expansive surface area and the incorporation of additional functional groups. The demonstrated superiority of j_0_ and k^0^ values underscores the advantageous characteristics of the composite modified electrode, emphasizing its effectiveness in promoting efficient electron transfer processes within the electrochemical system involving [Fe(CN)_6_]^3–/4–^.

### 3.3 Optimization of Experimental Conditions

#### 3.3.1 Effect of Electrolyte and pH

**Figure 5:**
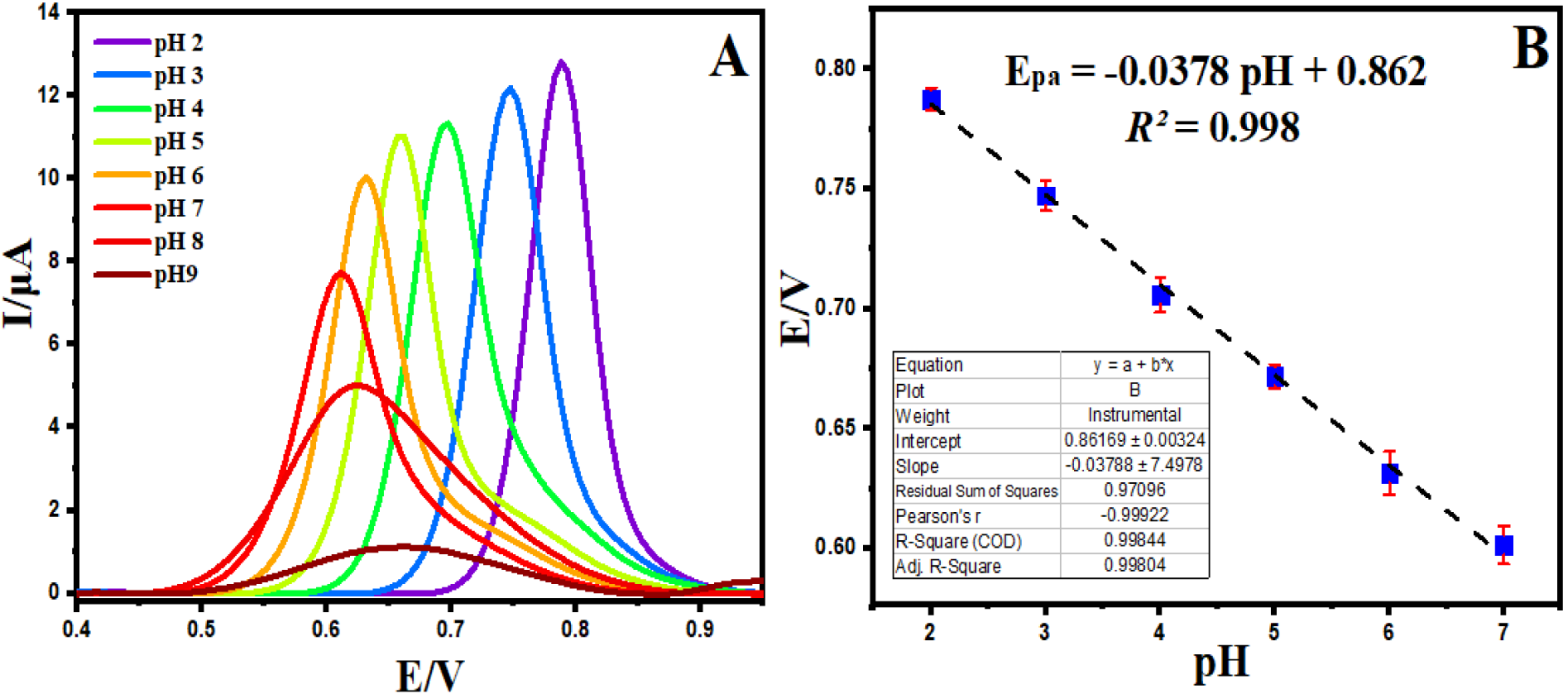
DPVs of 1 mM PLB at BPAC/NiFe_2_O_4_/MnCoFe-LDH/GCE in BR buffer of different pH values (A), and impact of pH on the *E*_pa_ values of PLB (B).

Supporting electrolytes play a crucial role in electrochemical investigations involving voltammetric techniques. They maintain a homogeneous electric field during the oxidation and reduction of the analyte molecule, ensuring consistent and reliable measurements. The pH of the solution also significantly impacts the signal of the analyte. To determine the optimal supporting electrolyte for PLB oxidation using BPAC/NiFe_2_O_4_/MnCoFe-LDH/GCE, differential pulse voltammograms (DPVs) were recorded in various supporting electrolytes: Britton–Robinson (BR), phosphate buffer saline (PBS), hydrochloric acid (HCl), potassium chloride (KCl), and sodium hydroxide (NaOH) (Figure S3). The results revealed that the supporting electrolyte significantly influenced the PLB oxidation current and potential peak. Among all the electrolytes tested, the BR buffer displayed the maximum peak current (I_pa_). Consequently, BR buffer was selected as the optimal supporting electrolyte for further experiments. The effect of pH on the PLB response in the BR buffer was subsequently investigated. Differential pulse voltammograms were recorded at various pH values, and the results are presented in Figure 5A. A well-defined peak was observed at pH 2.0, prompting its selection for further exploration. With an elevation in pH (2.0–7.0), the PLB potential peak values shifted toward less positive potentials (Figure 5B). Interestingly, the peak potential became pH-independent within this pH range. Figure 4C illustrates that the pH-independent peak potential exhibits a slope value of 0.037 V/pH. This slope value, which is half the Nernstian value (59 mV/pH), suggests the transfer of half the number of protons and electrons. The corresponding slope equation can be represented as E_p_ = (−0.0378 ± 7.497) pH + (0.862 ± 0.003) (R^2^ = 0.998).

#### 3.3.2 Effect of Concentration and Amount of Composite

In the quest to create an electrochemical sensor with superior performance for the determination of PLB, the determination conditions were meticulously optimized by systematically adjusting the quantity and concentration of the BPAC/NiFe_2_O_4_/MnCoFe-LDH composite. Through a series of experiments, the optimal conditions were determined to be a composite amount of 7 μL and a composite concentration of 1.5 M, as illustrated in Figure S4. Further details on the results of this section can be found in the Supporting Information file.

#### 3.3.3 Impact of Scan Rate

**Figure 6:**
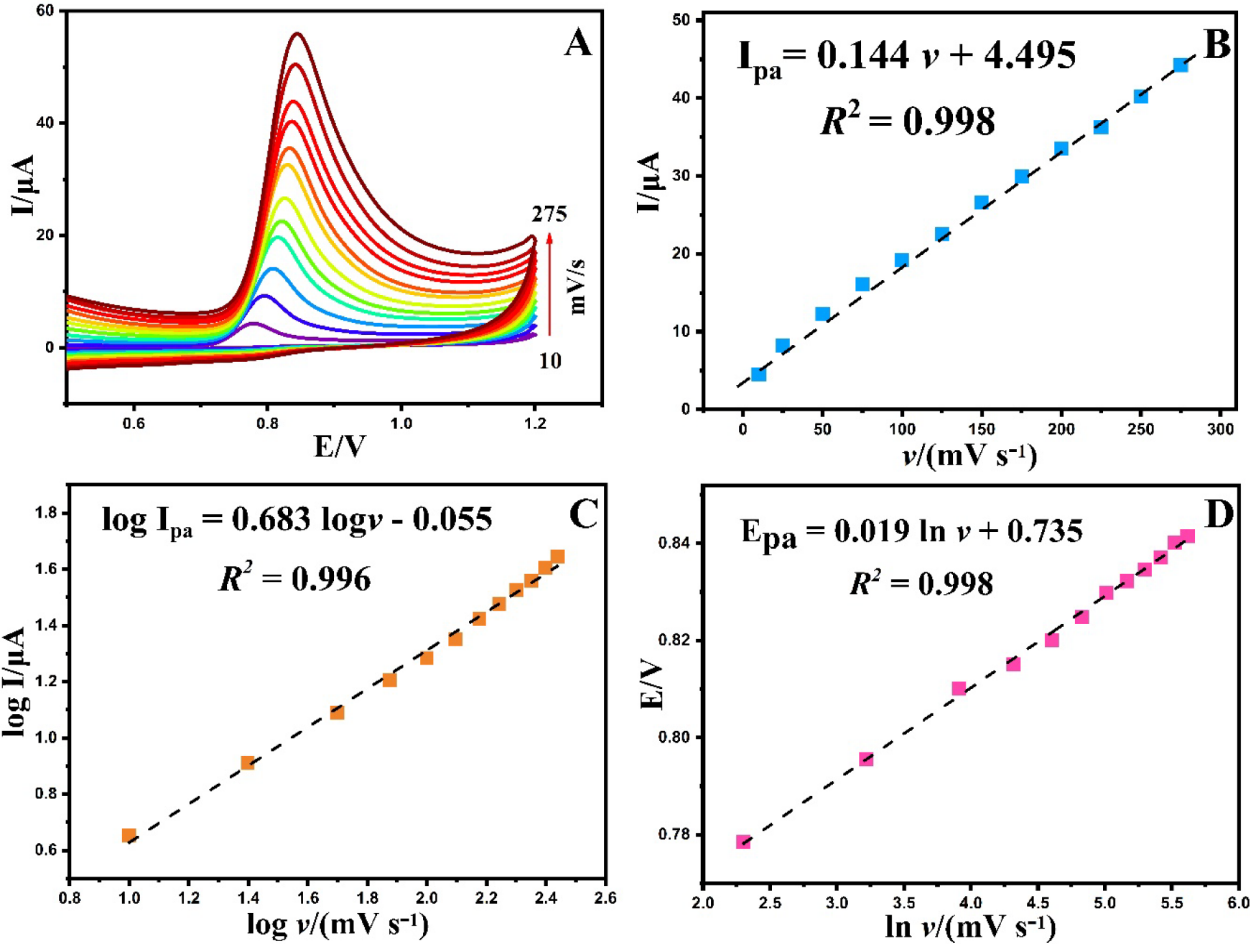
CVs recorded at 10–275 mV/s scan rates in BR buffer (pH = 2.0) with a 0.1 mM PLB (A), the linear dependence between the peak current and the scan rate (B), dependence of the logarithm of current peak vs log of scan rate (C), the linear correlation between the peak potential and the ln of the scan rate (D) using BPAC/NiFe_2_O_4_/MnCoFe-LDH/GCE.

In the realm of voltammetric measurements, a comprehensive examination of the scan rate assumes a pivotal role in unraveling the physicochemical attributes of the analyte molecule. In our exploration of the scan rate’s impact, we employed the cyclic voltammetry (CV) technique. The experimental setup involved conducting measurements on a 0.1 mM PLB solution at pH 2.0 in BR, with the scan rate varying from 0.01 to 0.275 V/s. The resulting voltammograms are presented in Figure 6A. It is noteworthy that elevating the scan rate led to an observable increase in peak current. Additionally, a subtle shift of the *E*_pa_ toward positive values was discerned, indicative of an irreversible process. The linear correlation between the oxidation peak currents and scan rates (I_p_ = (0.144 ± 0.002) υ + (4.495 ± 0.345), R^2^ = 0.998) highlights the absorption-controlled nature of the electron transfer behavior (Figure 6B). Furthermore, Figure 6C illustrates the logarithmic relationship between log I and log υ, expressed by log I = (0.683 ± 0.015) log υ – (0.055 ± 0.03) (R^2^ = 0.996). The slope of 0.683, observed between 0.5 and 1.0, aligns with the characteristic behavior of an adsorption-controlled process.

Figure S5 presents the Tafel plot alongside its corresponding voltammograms, illustrating the electro-oxidation of PLB at a scan rate of 100 mV s^–1^. Employing the slope of the Tafel plot, expressed as (2.3RT/n(1−α)F), the determined electron transfer coefficient (α) value was approximately 0.68. This observation confirms that the activation free energy curve for the irreversible electro-oxidation mechanism is asymmetric, as indicated by the nonsymmetrical nature of the obtained α value.

**Scheme 1:**
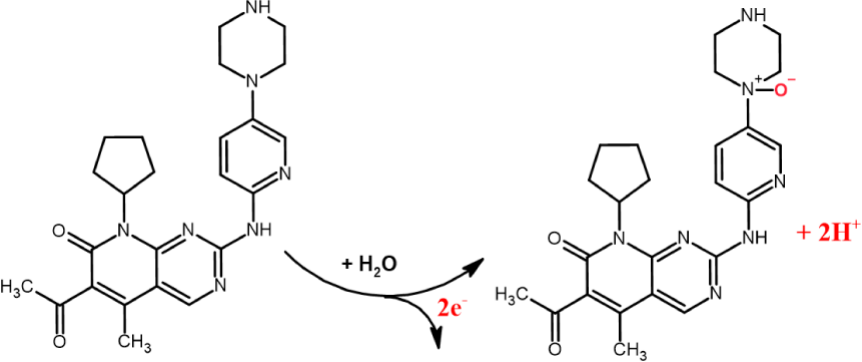
Proposed Mechanism for Electrooxidation of PLB at BPAC/NiFe_2_O_4_/MnCoFe-LDH/GCE

Furthermore, the examination of the potential peak in relation to the natural logarithm (ln) of the scan rate values (Figure 6D) yielded the following equation: *E*_p_ = (0.019 ± 2.89 × 10^–4^) + (0.735 ± 0.001) ln υ (R^2^=0.998). According to Laviron’s theory, the slope of *E*_p_ vs ln υ corresponds to RT/(αnF) for an irreversible electrode reaction process.^36^ Consequently, the calculation of the number of protons participating in this electrode reaction process yielded a value of 2.0. Given that the electron transfer number equates to the number of protons, it can be deduced that the electrochemical oxidation of PLB on the BPAC/NiFe_2_O_4_/MnCoFe-LDH/GCE entails a two-electron and two-proton transfer process. The possible electrochemical oxidation mechanism of PLB has been depicted in Scheme 1.^37^

#### 3.3.4 Effect of Accumulation Potential and Time

The process occurring at the electrode surface was identified as an adsorption-controlled mechanism. Consequently, the accumulation conditions were explored using DPV in a 0.1 M BR solution containing 1 mM PLB (Figure S6). The accumulation potential was systematically investigated by varying it from 0.1 to 1.1 V. The oxidation peak currents of PLB exhibited a gradual increase followed by a decrease when the potential exceeded 0.8 V. Hence, the optimum accumulation potential was determined to be 0.8 V (Figure S6A). Furthermore, the impact of deposition time on the peak current of PLB was studied within the range of 0 to 80 s. The oxidation peak current exhibited a gradual increase with an extended accumulation time up to 60 s. Beyond this duration, the peak current remained unchanged, indicating the saturated adsorption of PLB at the electrode surface (Figure S6B). For practical purposes, a 60 s accumulation period was selected.

### 3.4 Linear Range and Method Validation

**Figure 7:**
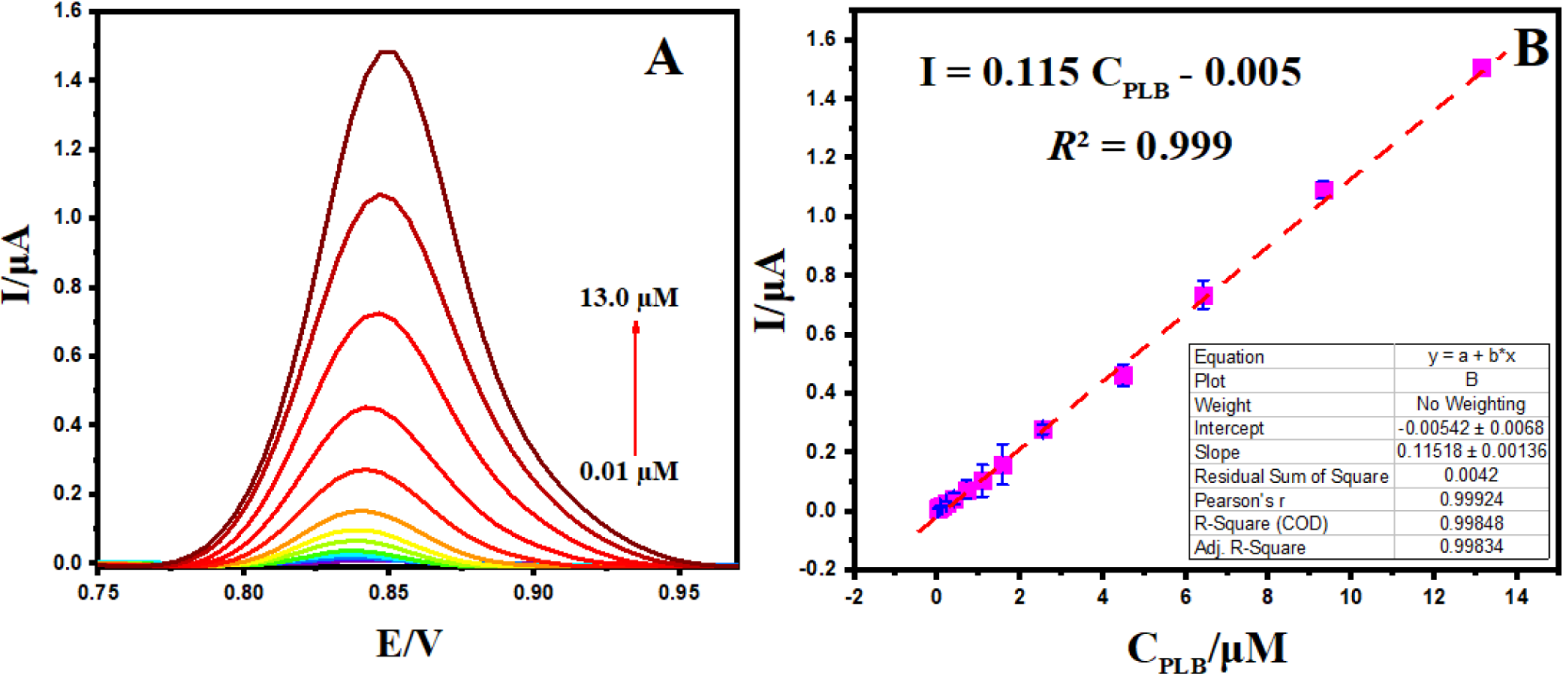
DPV responses of developed BPAC/NiFe_2_O_4_/MnCoFe-LDH/GCE for different concentrations of PLB (A) and the plot of I–C_PLB_ (B).

Differential Pulse Voltammetry (DPV) was employed to explore the linear range and the detection limit of PLB using BPAC/NiFe_2_O_4_/MnCoFe-LDH/GCE. DPV was chosen as the preferred electrochemical technique due to its adeptness in discriminating background current, providing superior peak resolution, and achieving low detection limits, thereby enabling effective detection of PLB. The results reveal that the oxidation of PLB takes place at 0.85 V vs Ag|AgCl, as depicted in Figure 7. Notably, the oxidation peak current (I_pa_) and the concentration of PLB (C) exhibit a well-defined linear relationship over the concentration range of 0.01–13.0 μM (Figure 7B). The corresponding linear regression equation was expressed as I (μA) = (0.115 ± 0.001) C (μM) – (0.005 ± 0.00) (R^2^ = 0.999), and the detection limit was determined to be 3.5 nM (S/N = 3). The excellent analytical performance can be attributed to the abundant binding sites and high electrochemical activity at the BPAC/NiFe_2_O_4_/MnCoFe-LDH nanocomposite.

The analytical performance of the TBPAC/NiFe_2_O_4_/MnCoFe-LDH/GCE was compared with previously reported PLB electrochemical sensors, and the results are summarized in Table 2. Notably, the proposed BPAC/NiFe2O4/MnCoFe-LDH-modified electrode demonstrated favorable analytical parameters when compared to existing works, including a broader dynamic range with a low LOD, and high repeatability. An additional advantage of the developed system lies in its ease of construction, environmental friendliness, and robust analytical performance.

**Table 2:** Comparison of Electrochemical Sensors for PLB

detection principle	electrochemical reduction	electrochemical oxidation	electrochemical oxidation
**electrode**	mercury electrode	NH_2_-MWCNT/GCE	TBPAC/NiFe_2_O_4_/MnCoFe-LDH/GCE
**voltammetric technique**	SWV	DPV	DPV
**linear range**	0.1–1 μM	0.2–2 μM	0.01–13.0 μM
**LOD**	8.8 × 10^–11^ M	4.82 × 10^–8^ M	3.5 × 10^–9^ M
**repeatability**	0.0282%	2.37%	1.3%
**recovery rate**	93% and 86.4%	99.9%	100.9% and 100.4%
**sample analysis**	human urine and plasma	tablet	human urine and tablet
**ref**	(9)	(38)	this work

**Figure 8:**
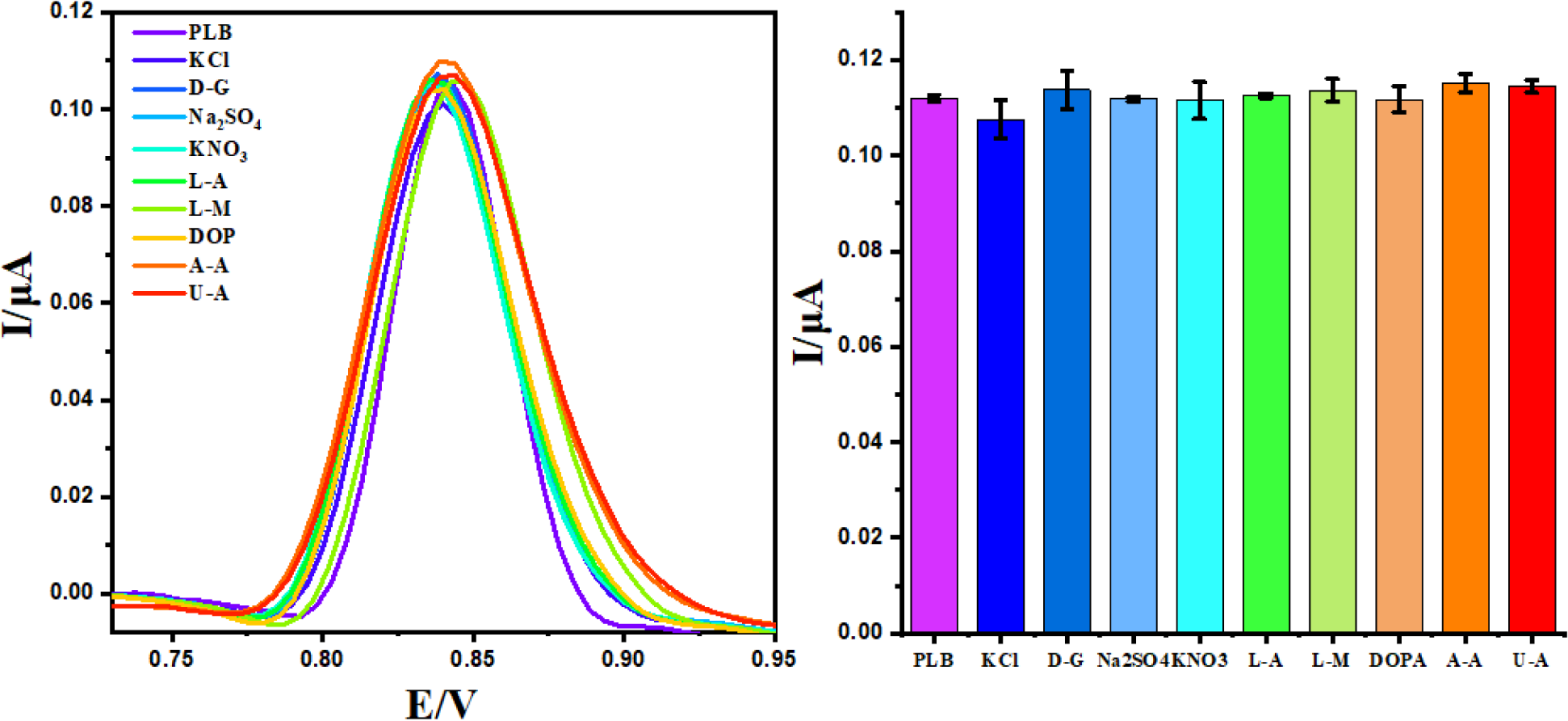
DPV curves and histogram of 1 μM PLB at on BPAC/NiFe_2_O_4_/MnCoFe-LDH GCE in the presence of different substances.

Selectivity is a crucial characteristic that must be taken into account when assessing the practical application of an electrochemical sensor. To assess the selectivity of BPAC/NiFe_2_O_4_/MnCoFe-LDH/GCE, anti-interference experiments were conducted. DPV responses were recorded for PLB (1 μM) sensing in the presence of a 100-fold concentration of potential interfering substances, including potassium chloride (KCl), d-glucose (D-G), sodium nitrate (Na_2_SO_4_), sodium sulfate (KNO_3_), l-arginine,^39^l-methionine,^40^ dopamine (DOPA), ascorbic acid (A-A), and uric acid (U-A). As depicted in Figure 8, a minor change in the peak current and potential was investigated, remaining within a permissible range, with a standard deviation of less than 1%. This outcome underscores the selectivity of the modified electrode for PLB detection, even in the presence of potentially interfering substances.

Repeatability and reproducibility investigations were conducted to assess the practicality of the developed BPAC/NiFe_2_O_4_/MnCoFe-LDH/GCE. Utilizing 11 successive cycles with a concentration of 1 μM of PLB under optimized experimental conditions, the developed sensor exhibited consistently repeatable peak current values, demonstrating minimal standard deviation (1.3% for *n* = 11) (Figure S7). Moreover, the reproducibility of the developed sensor was evaluated by implementing 9 different electrodes utilizing the same modification process. Remarkably, the system preserved the consistency of the current response with a nominal variance in the peak current signal and a low RSD (2.2% for *n* = 9) (Figure S8). These results underscore the outstanding consistency in repeatability and reproducibility observed in the developed BPAC/NiFe_2_O_4_/MnCoFe-LDH/GCE, affirming its reliability for consistent and accurate PLB detection.

To validate the practicality and feasibility of the electrochemical sensor for real-world sample analysis, the developed BPAC/NiFe_2_O_4_/MnCoFe-LDH/GCE was applied to quantify the PLB content in both human urine and tablets. Under optimized conditions, the standard addition method was employed to perform sample recovery experiments for the accurate determination of PLB. This approach ensures a robust evaluation of the sensor’s performance in complex matrices, such as biological samples and pharmaceutical formulations. The obtained percentage recoveries were in the range of 98.5–102.9%, with RSD values lower than 3% (Table 3), proving the accuracy and reliability of the developed sensor. In addition to evaluating the percentage recovery of the analyte across the range of the assay, the linearity of the relationship between estimated and actual concentrations for real samples was further assessed (Figure S9). For human urine samples, the linear regression analysis yielded a slope of 1.02 (R^2^ = 0.999), indicating an excellent correlation between estimated and actual concentrations. Similarly, for tablet samples, the slope was determined to be 0.97 (R^2^ = 1), further confirming the accuracy of the assay. These results aligned with the statistically preferred criterion, demonstrating the robustness and reliability of the analytical method.

**Table 3:** Analysis of PLB in Human Urine and Tablets

sample	added (μM)	detected (μM)^a^	RSD (%)	recovery (%)
human urine	1.0	1.02	1.9	102.3
	2.0	1.97	2.8	98.7
	3.0	3.05	2.0	101.8
tablet	1.0	1.03	2.1	102.9
	2.0	1.99	0.5	99.9
	3.0	2.96	1.2	99.9

a Average of three replicate measurements.

## 4 Conclusion

In conclusion, the presented study has successfully developed an ultrasensitive electrochemical sensor for the detection of Palbociclib (PLB) based on a novel composite material, BPAC/NiFe_2_O_4_/MnCoFe-LDH, modified onto a GCE. The synthesized composite exhibited excellent electrocatalytic activity, sensitivity, and selectivity toward PLB, providing a broad linear concentration range of 0.01–13.0 μM, a low LOD of 3.5 nM, and remarkable repeatability and reproducibility. The synergistic effect between BPAC, NiFe_2_O_4_, and MnCoFe-LDH was responsible for the excellent analytical performance of the fabricated sensor. Moreover, the prepared sensor demonstrated its practical applicability through accurate PLB determination in both human urine and pharmaceutical formulations, showcasing its potential for real-world sample analysis. The presented work contributes to the advancement of electrochemical sensors for pharmaceutical analysis, with the developed sensor exhibiting not only superior analytical performance but also ease of construction and environmental friendliness. This research opens avenues for further exploration and optimization of electrochemical sensing platforms for other pharmaceutical compounds, emphasizing the significance of such sensors in clinical and pharmaceutical research.

## Supporting Information Available

Additional experimental details include materials, reagents, apparatus, synthesis of studied nanocomposites, relevant equations, details on the effect of concentration and amount of composite, cyclic voltammogram, and differential pulse voltammograms (PDF)
