# Supplementary material for: An Ultrasensitive Electrochemical Sensor Using Banana Peel Activated Carbon/NiFe2O4/MnCoFe-LDH Nanocomposites for Anticancer Drug Determination
Source: ACS Omega. 2024 Jun 12;9(25):27446–57. doi: 10.1021/acsomega.4c02460 (PMC11209681; doi:10.1021/acsomega.4c02460)
Supplement: Supplementary file 1 — ao4c02460_si_001.pdf [file ao4c02460_si_001.pdf]

Supporting Information

Ultrasensitive electrochemical Sensor using banana peel activated carbon/$NiFe_2O_4$/MnCoFe-LDH nanocomposites for anticancer drug determination

Nevin Erk [a*], ***Wiem Bouali*** [a,b*], ***Asena Ayse Genc*** [a,b], ***Qamar Salamat*** [c], ***and Mustafa Soylak*** [c,d,e]

[a] Ankara University, Faculty of Pharmacy, Department of Analytical Chemistry, 06560 Ankara, Turkey

[b] Ankara University, The graduate school of the health sciences, 06110 Ankara, Turkey

[c] Erciyes University, Faculty of Sciences, Department of Chemistry, 38039, Kayseri, Turkey

[d] Technology Research & Application Center (TAUM), Erciyes University, 38039, Kayseri, Turkey

[e] Turkish Academy of Sciences (TUBA), Cankaya, Ankara, Turkey

Email corresponding author: erk@pharmacy.ankara.edu.tr

wbouali@ankara.edu.tr

Materials and Reagents

In this study, Glucose (99.5 %), L-arginine (98.0 %), L-methionine, sodium hydroxide, potassium hexacyanoferrate (III) ($K_3Fe(CN)_6$, 99.5 %), hydrochloric acid, sodium Acetate, ascorbic acid, uric acid (99.0 %), acetic acid, potassium chloride, sodium phosphate, sodium sulfate, potassium chloride, sodium sulfate, were purchased from Sigma Aldrich Co. (https://www.sigmaaldrich.com, Germany). Britton-Robinson buffer was made of boric acid, phosphoric acid, potassium chloride, and acetic acid solutions. The stock solution of Palbociclib was prepared in methanol: water (1:1). All chemical compounds were analytical grade and used without additional refinement.

Apparatus

Voltammetric experiments were carried out using AUTO LAB system with PGSTAT204 electrochemical workstation (Metrohm Inc., Switzerland) with a glassy carbon electrode system in a one-compartment of 10 mL electrochemical cell. All electrochemical measurements were performed at 25 °C unless otherwise specified.

Synthesis of banana peel activated carbon

The preparation of banana peel activated carbon (BPAC) was performed according to previous research [1], with some modifications as follows: First, they were thoroughly washed with tap water and distilled water to remove the impurities. Afterward, they were cut into small pieces (1.0×1.0 cm) before being dried in the oven at 100 ◦ C for 20 h. The dried banana peels were then crushed with a mortar and passed through a sieve with a mesh size of 200 µm. The obtained powder was then placed in a muffle furnace at a carbonized temperature of 550 ◦ C for 1.5 h. After cooling the carbonized sample, it was subjected to a potassium hydroxide 45% aqueous solution (weight ratio 1:5 of carbonized material to potassium hydroxide), followed by stirring for 12 h on a magnetic stirrer. After that, the obtained material was placed at room temperature for 24 h before being dried in an oven at 120 °C for 72 h. In the next step, the dried sample was then placed again in a muffle furnace at 750 °C for 1 h to be activated. Finally, the sample was cooled to room temperature, and then the obtained activated carbon was washed sequentially with 1 M hydrochloric acid and distilled water up to a neutral pH, as well as removing excess potassium hydroxide compounds. Eventually, the final product (BPAC) was achieved by drying in an oven at 110 ◦ C for 12 h.

Synthesis of $NiFe_2O_4$ nanoparticles

2.5 mmol $Ni(NO_3)_2 \cdot 6H_2O$, 2.5 mmol $Fe(NO_3)_3 \cdot 9H_2O$, 8.5 mmol 1,3,5-trimesic acid, and 3.5 mmol 2-methylimidazole were dispersed in 60 mL DMF, followed by stirring for 30 min until creating a homogeneous solution. The resulting mixture was transferred to a 60-mL Teflon container, placed in an autoclave, and heated in an oven at 170 ◦C for 24 h. After cooling to room temperature, the synthesized material was thoroughly washed with ethanol, dried in a vacuum oven overnight, and then calcined at 750 °C for 2.5 h in a muffle furnace to obtain $NiFe_2O_4$ [2].

The Randles-Sevcik equation:

$$I = (2.69 \times 10^5)\, n^{\frac{3}{2}} A\, D^{\frac{1}{2}}\, v^{\frac{1}{2}}\, C_0 \qquad (S1)$$

A represents the electrode area in cm^2, D shows the diffusion coefficient (cm^2/s), n is the number of electrodes (n = 1), v exhibits the potential scan rate (V/s), and C_0 is the concentration (mol/cm^3).

$$R_{ct} = \frac{RT}{F^2\, C A k^0} \qquad (S2)$$

$$R_{ct} = \frac{RT}{n\, F\, A\, j_0} \qquad (S3)$$

k^0 represents the standard heterogeneous electron transfer rate constant ($cm\ s^{-1}$), j_0 is the exchange current density ($A\ cm^{-2}$), R stands for the universal gas constant ($8.314\ J\ K^{-1}\ mol^{-1}$), T is the temperature (298.15 K), F is the Faraday constant ($96485\ C\ mol^{-1}$), R_{ct} is the electron transfer resistance (Ω), A is the electrode surface area (cm^2), n is the number of electrons transferred, and C is the concentration of the $[Fe(CN)^6]^{3-/4-}$ solution ($5 \times 10^{-6}\ mol\ cm^{-3}$).

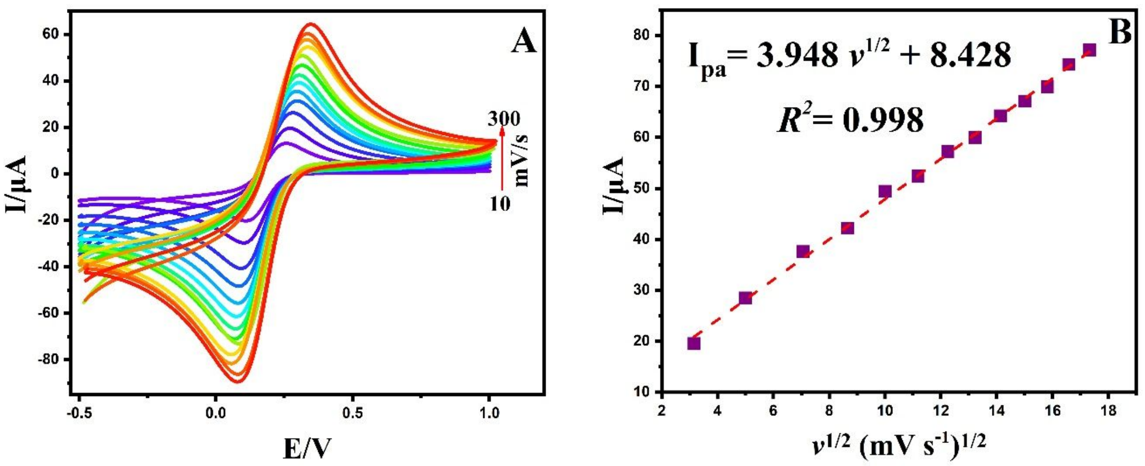

Figure S1. The recorded CV curves (A), and the relationship between I_{pa} vs. $v^{1/2}$ (B) on the bare GCE at various scan rates in 5.0 mM $[Fe(CN)_6]^{3-/4-}$ and 0.1 M KCl.

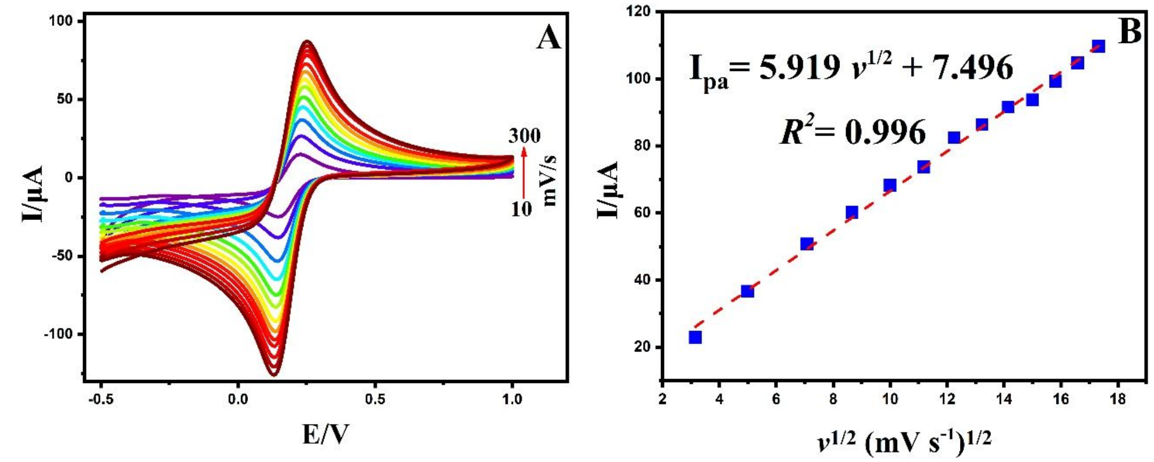

Figure S2. The recorded CV curves (A), and the relationship between I_{pa} vs. $v^{1/2}$ (B) on the BPAC/$NiFe_2O_4$/MnCoFe-LDH/GCE at various scan rates in 5.0 mM $[Fe(CN)_6]^{3-/4-}$ and 0.1 M KCl.

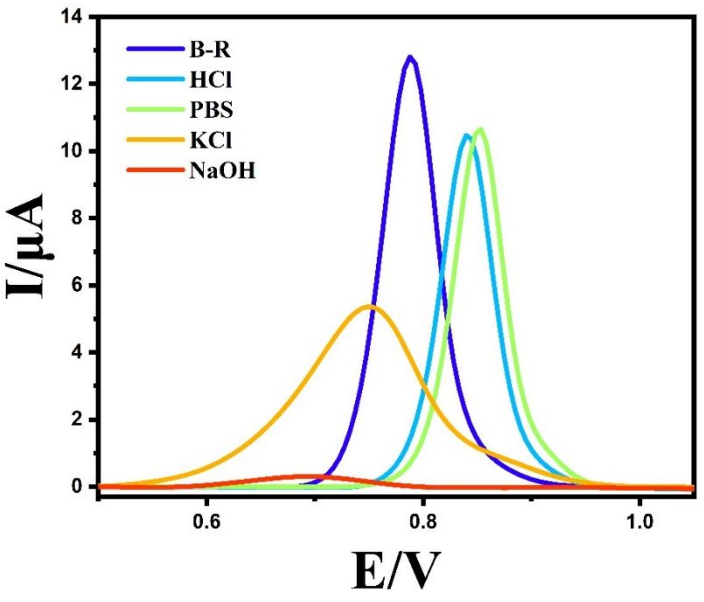

Figure S3. Influence of supporting electrolyte on the oxidation peak currents of 0.1 mM PLB at the surface of BPAC/$NiFe_2O_4$/MnCoFe-LDH/GCE.

Effect of concentration and amount of composite

In the initial step of the optimization process, different amounts of BPAC/$NiFe_2O_4$/MnCoFe-LDH (ranging from 3.0 µL to 9.0 µL) onto the GCE's surface in a 0.1 M BR buffer (pH = 2.0). As depicted in Figure S4A, an increase in the composite amounts up to 7 µL significantly enhanced the oxidation peak current, indicating improved surface area and catalytic sites. Nevertheless, as the amount of BPAC/$NiFe_2O_4$/MnCoFe-LDH dispersion further increased, the peak current of PLB exhibited a noticeable decrease.

The next step in the optimization process involved evaluating PLB signals at different concentrations of the BPAC/$NiFe_2O_4$/MnCoFe-LDH composite, ranging from 0.5 M to 2.0 M, for the modification of the GCE. As depicted in Figure S4A, there was an observable increase in the electro-oxidation current of PLB with 1.5M concentrations of the BPAC/$NiFe_2O_4$/MnCoFe-LDH composite. However, beyond a composite concentration of 1.5 M the peak intensity started to decrease. Consequently, the optimal composite concentration for determining PLB was identified as 1.5 M.

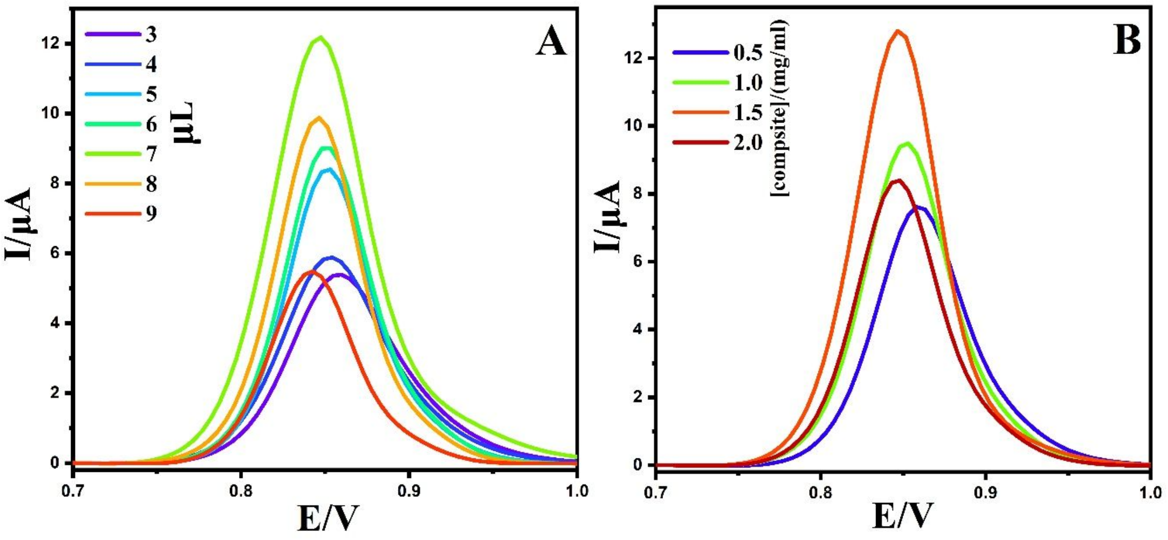

Figure S4. Influence of the amount (A), and the concentration (B) of BPAC/$NiFe_2O_4$/MnCoFe-LDH composite on the oxidation peak currents of 0. 1 mM PLB.

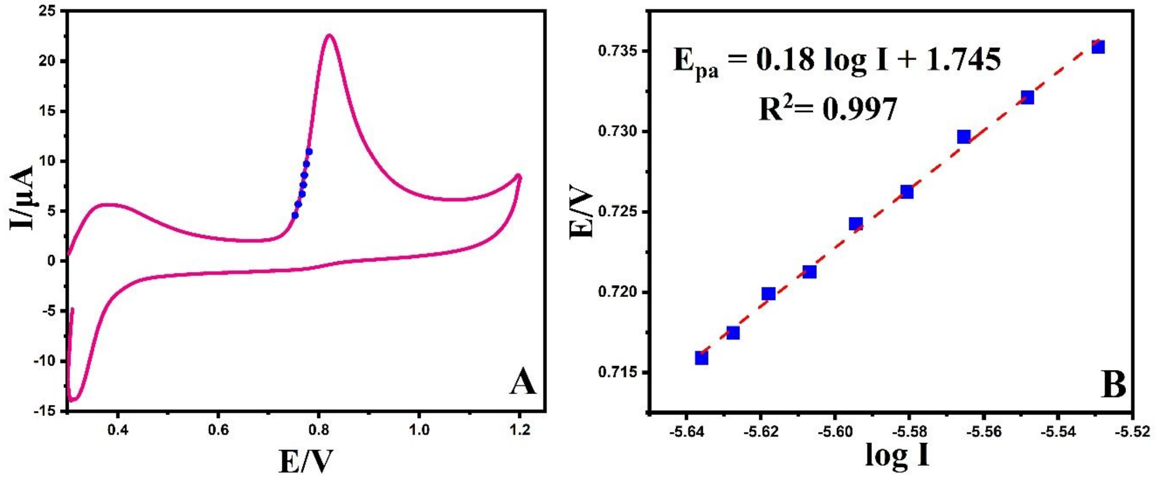

Figure S5. DPV (A) of PLB (1 mM) at the surface of BPAC/$NiFe_2O_4$/MnCoFe-LDH/GCE in BR buffer (pH 2.0) with a scan rate of 100.0 mV/s and the relationship between Log Ipa vs. Epa (B).

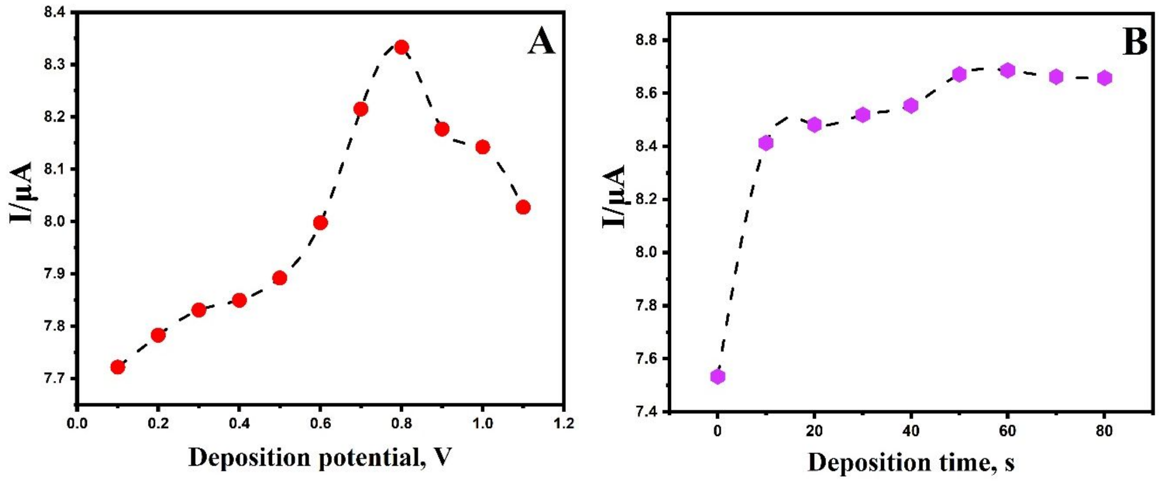

Figure S6. Effect of deposition potential (A), and deposition time (B); on peak current of 0.1 mM PLB at the BPAC/$NiFe_2O_4$/MnCoFe-LDH/GCE surface.

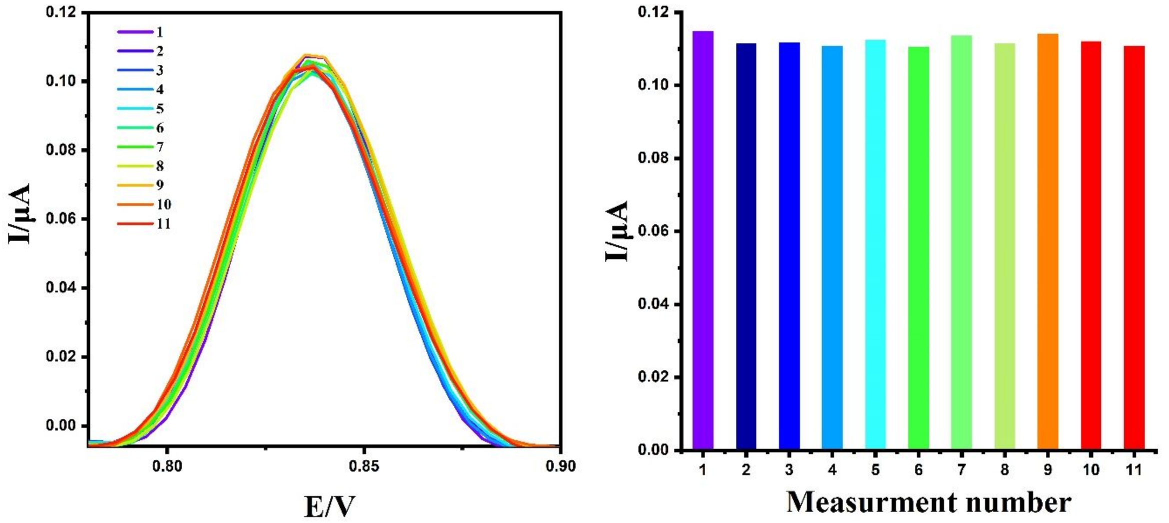

Figure S7. DPV curves and histogram of repeatability of BPAC/$NiFe_2O_4$/MnCoFe-LDH/GCE in 1.0 μM PLB (B-R buffer, pH 2.0).

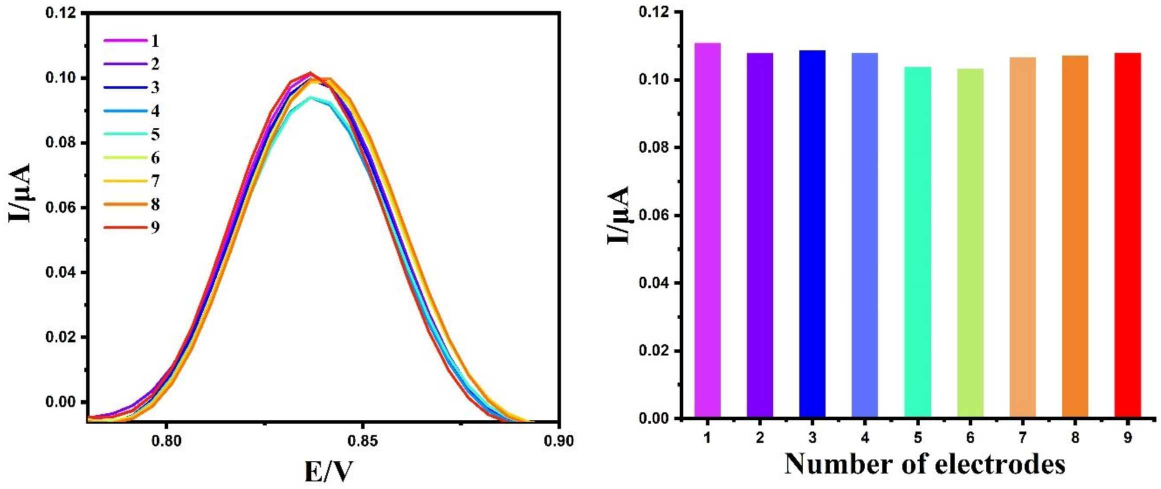

Figure S8. DPV curves and histogram of reproducibility of BPAC/$NiFe_2O_4$/MnCoFe-LDH/GCE in 1.0 µM PLB (B-R buffer, pH 2.0).

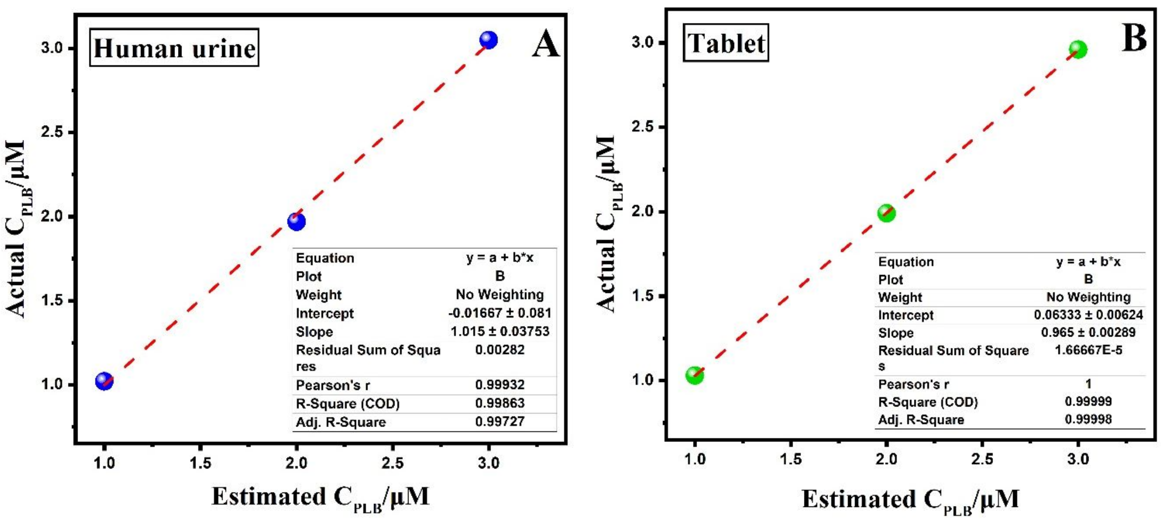

Figure S9. Correlation between the estimated and actual concentrations of PLB in human urine (A) and tablet (B).

References

(1) Ahmadi, S.; Ganjidoust, H. Using banana peel waste to synthesize BPAC/ZnO nanocomposite for photocatalytic degradation of Acid Blue 25: Influential parameters, mineralization, biodegradability studies. *Journal of Environmental Chemical Engineering* **2021**, *9* (5), 106010.

(2) Chen, Y.; Fang, S.; Sun, L.; Xu, F.; Wang, M.; Zhang, J.; Mu, X.; Wang, X.; Wang, P.; Liu, J. Hierarchical NiFe2O4-NiAl-LDH arrays immobilized on activated carbon cloth for bifunctional application on high-performance supercapacitors and solar steam generation. *Sustainable Materials and Technologies* **2022**, *33*, e00500.
